# Pre and post-analytical guidelines for the microscopic diagnosis of melanoma: recommendations from the Brazilian Society of Pathology^☆^

**DOI:** 10.1016/j.abd.2025.501139

**Published:** 2025-07-03

**Authors:** José Cândido Caldeira Xavier-Júnior, Karina Munhoz de Paula Alves Coelho, Mariana Petaccia de Macedo, Rute Facchini Lellis, Nathanael de Freitas Pinheiro Junior, Robledo Fonseca Rocha, Dermatopathology Committee of the Brazilian Society of Pathology

**Affiliations:** a Instituto de Patologia de Araçatuba, Araçatuba, SP, Brazil; b Faculty of Medicine, Centro Universitário Católico Unisalesiano, Araçatuba, SP, Brazil; c Postgraduate Program in Pathology, Faculty of Medicine, Universidade Estadual Paulista, Botucatu, SP, Brazil; d CEDAP, Centro de Diagnósticos Anátomo-Patológicos, Joinville, SC, Brazil; e Instituto Nacional de Ciência e Tecnologia em Biologia do Câncer Infantil e Oncologia Pediátrica ‒ INCT BioOncoPed, Brazil; f Department of Pathology, Rede D’Or/São Luiz Hospital, São Paulo, SP, Brazil; g Santa Casa de Misericórdia de São Paulo, São Paulo, SP, Brazil; h Imagepat Anatomia Patológica Ltda, Salvador, BA, Brazil; i Centro de Ciências da Saúde, Universidade Federal de Roraima, Boa Vista, RR, Brazil; j Laboratório de Patologia de Roraima, Boa Vista, RR, Brazil

**Keywords:** Dermatology, Guideline, Melanoma, Microscopy, Pathology

## Abstract

The guidelines project of the Brazilian Society of Pathology aims to disseminate recommendations for pathologists, surgeons, and clinicians, based on solid data from the literature and through adaptations of international guidelines to the reality of Brazilian physicians. This article is the result of the efforts of a group of pathologists, members of the Dermatopathology Committee of the Brazilian Society of Pathology, focused on melanocytic diseases, who, through topics, established pertinent recommendations for clinicians and surgeons for the accurate diagnosis of melanocytic lesions suspected of melanoma. This article aims to clarify the best way to perform excision in cases of suspected melanocytic lesions, as well as the pre-analytical care related to the material, how to interpret the anatomopathological report, and the situations in which immunohistochemical and molecular studies can be auxiliary tools for diagnosis and/or therapy.

## Introduction

According to data from the Global Cancer Observatory (GLOBOCAN), in 2022, 331,647 new cases and 58,645 deaths from cutaneous melanoma were identified^1^; the primary skin tumor is the most lethal and there is a tendency for increased mortality from this neoplasm in the elderly Brazilian population.^2^ Moreover, national data, in agreement with the international literature, indicate an increase in the incidence of melanoma cases in a time series,^3^, ^4^ with stability of histological subtypes that show early invasive behavior, which corresponds to a considerable fraction of specific mortality.^3^ Patients with clinically suspicious pigmented skin lesions should preferably undergo (whenever possible) surgical excision (excisional biopsy),^5^ since the diagnosis remains anatomopathological, despite all the advances in molecular pathology and *in-vivo* imaging tools regarding the knowledge of these neoplasms.^6^ As is also the case with most malignant neoplasms in other sites, an accurate early diagnosis of cutaneous melanoma is associated with a better prognosis.^7^, ^8^ In addition, the creation of standardized protocols for collection, preparation, analysis, and structured reports is essential for the correct monitoring of these patients^9^ and to ensure the integrity of the material for possible complementary studies (immunohistochemical and molecular ones), when applicable.

It is worth noting that this guideline was developed by the Brazilian Society of Pathology (SBP, *Sociedade Brasileira de Patologia*), which created a working group of experts in the area to evaluate the literature, Brazilian legislation, classic books, and international recommendations to propose a protocol oriented to the reality of health care in the country. These recommendations will be published in two articles, with similar and complementary content. The present article was written with a focus on clinicians and surgeons, while another article, aimed at pathologists, will be published in the journal Surgical and Experimental Pathology, of SBP. The present article aims to clarify the best way to perform excision in cases of suspicious melanocytic lesions, as well as the pre-analytical care with the material, how to interpret the anatomopathological report, and the situations in which immunohistochemical and molecular studies can be auxiliary tools for diagnosis. Table 1 shows the list of all recommendations in this article.

**Table 1:** Summary of recommendations.

Recommendation 1	In the case of suspected melanoma, excisional biopsy with exigous margins (1‒3 mm) should be the preferred approach.
Recommendation 2	After removal, the biopsy/surgical specimen should be immediately submerged in an appropriate container containing buffered formalin (10% buffered formaldehyde) in a volume approximately 10 to 20 times the sample size.
Recommendation 3	Clinical information, especially patient age and sex, as well as lesion location and size, are essential data for the accurate microscopic diagnosis of melanocytic lesions.
Recommendation 4	Whenever possible, include all the material sent in case of lesions suspected of melanoma. Clinicians and surgeons are advised to verify whether this recommendation was followed by carefully reading the macroscopy section of the anatomopathological report.
Recommendation 5	Immunohistochemical studies are not essential for the microscopic diagnosis of melanoma, being reserved for selected cases, indicated by the pathologist in the report, often through comments and notes.
Recommendation 6	Perioperative examination is contraindicated for both diagnosis and margin evaluation of melanocytic lesions.
Recommendation 7	Perioperative examination is contraindicated for sentinel lymph node evaluation in cases of cutaneous melanoma.
Recommendation 8	Molecular testing to detect mutations in the *BRAF* gene is recommended in the scenario of patients with high-risk stage 2, 3 and 4 melanoma, using a sample with adequate neoplastic representation and a molecular technique duly validated by the laboratory.

## Surgical procedure

Providing an appropriate biopsy and a pertinent clinical history is essential for the diagnosis and accurate prognosis of melanoma.^5^ In cases of lesions suspected of melanoma, an excisional biopsy with exiguous free margins should be the preferred approach. Occasionally, an incisional biopsy may be considered the first approach in very large lesions or in lesions located in regions of aesthetic or functional interest. Shaving biopsy is expressly contraindicated because it may add difficulties to the histopathological diagnosis and, in the future, compromise the correct determination of the Breslow index (thickness).^10^, ^11^, ^12^ The use of the Mohs surgery technique is not validated for the approach to melanocytic lesions and may also compromise the accurate diagnosis of these lesions.^13^

**Figure 1:**
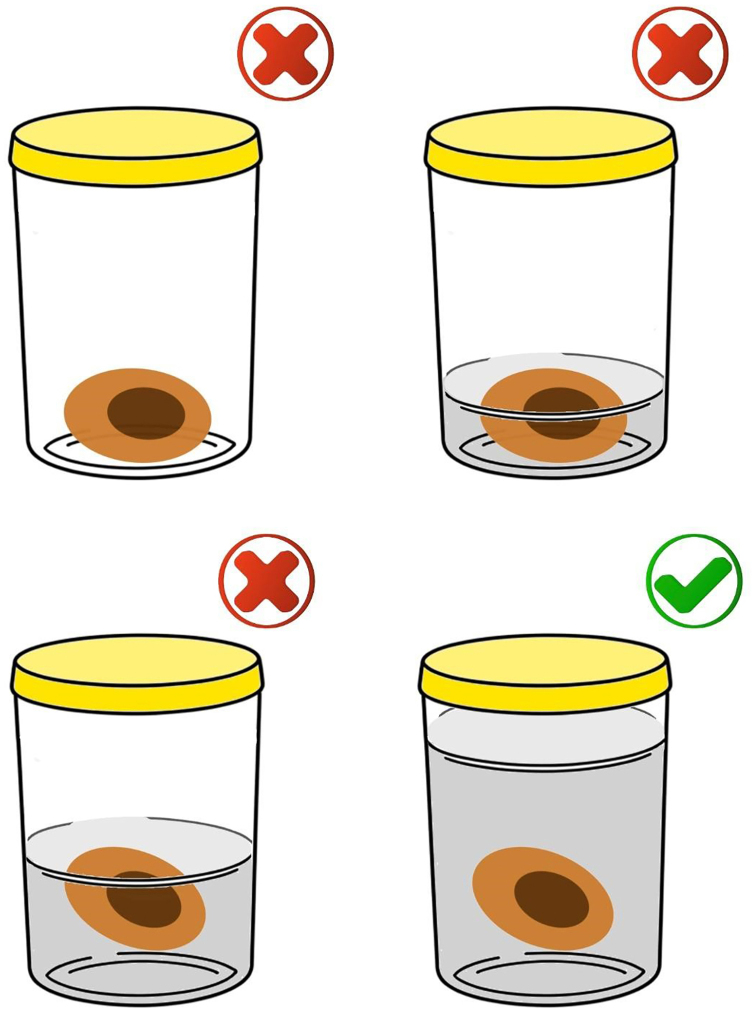
The correct way to store the specimen is using a volume of 10% buffered formalin greater than 10‒20 times the volume of the specimen (bottom right image). It is not acceptable to send the material without formalin, in a volume that does not cover the specimen in its entirety or in a volume slightly greater than the volume of the specimen.

After the procedure, the biopsy/surgical specimen must be immediately submerged in a clean, transparent, wide-mouthed, well-sealed, and accurately identified container containing buffered formalin (10% buffered formaldehyde) in a volume approximately 10 to 20 times the size of the specimen (Fig. 1). The ideal fixation time may vary between six and 72 hours. It is expressly contraindicated to send specimens/biopsies in other media such as petroleum jelly, alcohol, or saline solution. All of these measures are important to maintain the morphological integrity of the tissue, as well as the proteins and genetic material of the tumor cells; potential future targets of immunohistochemical and molecular studies, respectively.^11^ To ensure that the formalin used is buffered, direct communication with the person in charge of the hospital unit or with the partner pathology laboratory is suggested. To prevent excessive fixation of the materials, the ideal is not to perform surgical excision (excisional biopsy) on the eve of holidays, or even in the late afternoon on Fridays, if the partner laboratory does not have processing hours on Saturday. Avoid performing surgical excision in an institution/clinic that does not have agile flow logistics for sending samples for processing and analysis to prevent long periods of exposure to formalin and material overfixation. It is important to highlight the impossibility of processing and diagnosing skin lesions on the same day as the lesion is removed since the minimum time for fixation and histological processing must be taken into account. It is also essential that only one excision specimen be placed in each container to allow correct lesion identification with the final histopathological diagnosis.

Handling of the surgical specimen after removal must be careful to prevent causing crushing artifacts.^14^ The use of electrocautery should be cautious, since, if excessive, it may impair the assessment of the lesion edges.^15^

**Recommendation 1**: In cases of suspected melanoma, excisional biopsy with exiguous margins (1-3 mm) should be the preferred approach.

**Recommendation 2**: After removal, the biopsy/surgical specimen should be immediately submerged in an appropriate container containing buffered formalin (10% buffered formaldehyde) in a volume approximately 10 to 20 times the sample size.

## Request for anatomopathological examination

In the absence of a specific request form from the laboratory, the request may be performed using a prescription form containing the physician's identification, date and minimum patient identification information (always consider at least three parameters, for example, full name, date of birth and Social Security Number or other identification document).^16^

**Figure 2:**
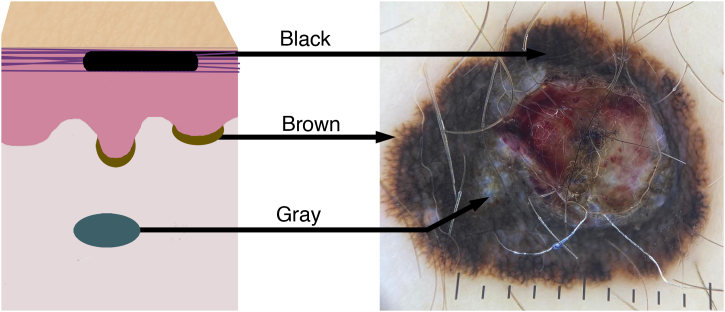
Color variation in melanocytic lesions reflects the location of the melanin pigment in the skin. The schematic drawing shows the correspondence of the colors/tones of melanoma in the dermoscopic examination according to the melanin deposit: black in the stratum corneum, brown in the basal layer of the epidermis, and bluish/gray in the dermis.

It is essential to send the clinical history and dermatological description of the lesion (Fig. 2), its evolution, results of complementary exams and diagnostic hypotheses, as they are part of the prerequisites for a full report and may influence the diagnostic decision in the case of melanocytic lesions. Sex, location of the lesion and patient age are the minimum data for microscopic analysis without which a conclusive and adequate diagnosis is at risk.^8^, ^17^, ^18^

**Figure 3:**
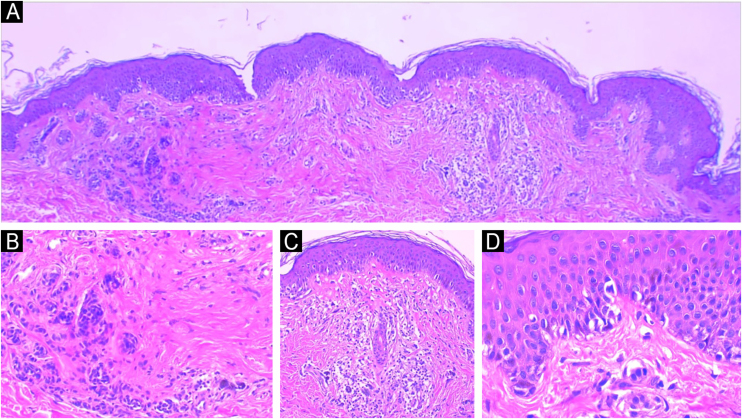
Traumatized nevi are a good example of the need for clinical-pathological correlation. See the panoramic image of a nevus on the thigh of a 44-year-old female patient. The remaining nevus can be seen on the left, the central fibrosis and the dermal inflammatory infiltrate with melanophages on the right (A) Hematoxylin & eosin ×40. In detail, the remaining intradermal nevus and the fibrosis parallel to the direction of the epidermis, Hematoxylin & eosin ×200 (B). In detail, the inflammatory infiltrate with melanophages, Hematoxylin & eosin ×200 (C). Presence of atypical proliferated junctional melanocytes, a common finding in traumatized melanocytic lesions, Hematoxylin & eosin ×400.

**Figure 4:**
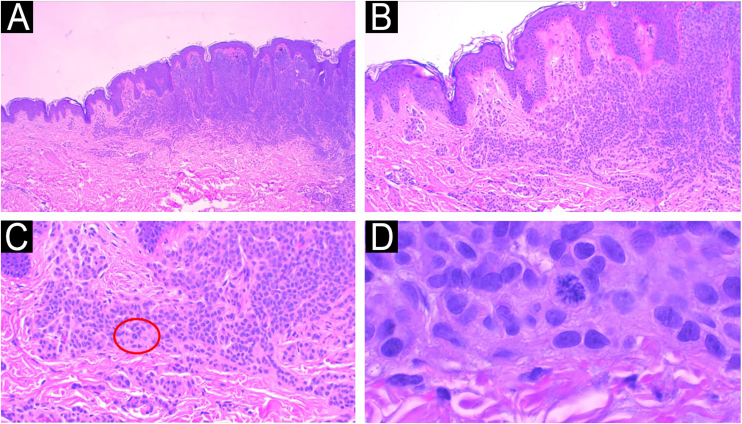
Another example of the importance of anatomical-clinical correlation: intradermal melanocytic nevus removed for aesthetic purposes from a 14-year-old female patient. Panoramic view of the intradermal nevus (A), Hematoxylin & eosin ×40. Melanocytes arranged in nests with maturation to depth (B) Hematoxylin & eosin ×100 and no signs of atypia (C) Hematoxylin & eosin ×200. The presence of atypical deep mitosis in the center of the image, which does not change the diagnosis, Hematoxylin & eosin ×1000.

Among the most relevant data for the analysis of a melanocytic lesion are: a) Age: Age range affects the interpretation of microscopic findings. For example, the same microscopic findings that would lead to the diagnosis of acral melanoma in an elderly patient may not characterize malignancy in a pediatric patient. b) Location: Benign melanocytic lesions located in so-called “special sites” (e.g., scalp, genitalia, breast line, auricule, and flexural regions) may present microscopic and dermoscopic findings simulating malignancy that would not be admissible in other anatomical locations. c) Clinical description of the lesion: ABCDE rule (asymmetry, borders, color, diameter, and evolution), as well as any other relevant information (e.g., increase in size, changes in characteristics, occurrence of trauma, results of previous biopsies, appearance of ulceration, mainly). d) Clinical history: Pregnancy,^19^ personal and family history of melanoma and dysplastic nevi. e) Other tests: Description of dermatoscopy and, if applicable, other complementary tests (e.g., confocal microscopy and germline genetic testing)^6^, ^20^, ^21^ (Figure 3, Figure 4).

**Recommendation 3**: Clinical information, especially patient age and sex, as well as lesion location and size, are essential data for the accurate microscopic diagnosis of melanocytic lesions.

## Pre-analytical laboratory evaluation

**Figure 5:**
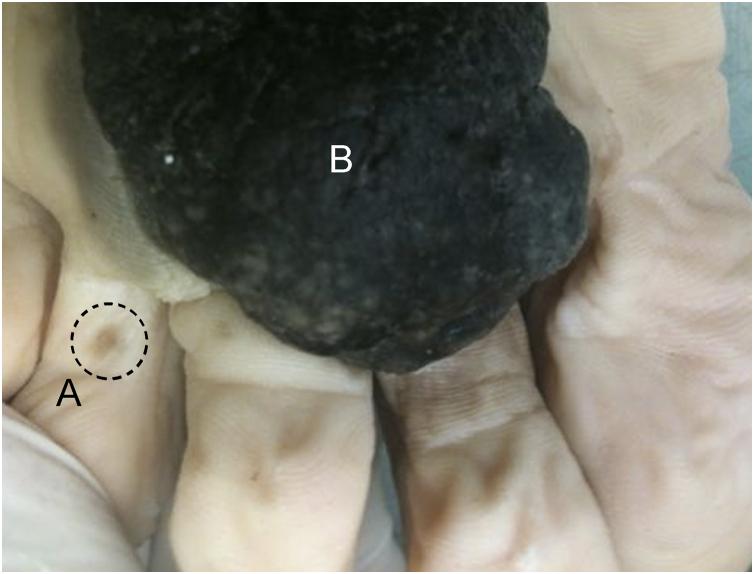
Macroscopic image of acral melanoma with a small discontinuous lesion (A) and a large tumor component (B). The identification of satellites/microsatellites/cutaneous metastases influences staging and can occur through detailed macroscopic examination.

The macroscopic description consists of measuring the skin fragment received and a direct but detailed description of the identified lesion (Fig. 5). Every anatomopathological report includes the macroscopic description, which is usually found just below the clinical information. This allows correlation with data from the surgical specimen sent. Ideally, the specimen should be included in its entirety. In cases of very large and clearly advanced lesions (e.g. amputation products with evident bone infiltration), detailed sampling may be an option. To verify whether all the material has been included, one should simply read the statement at the end of the macroscopy. It is also possible to verify in the macroscopic description how many paraffin blocks resulted from the material inclusion, which must be identified.

See below, for instance, the macroscopic description of melanoma, in which it is highlighted that all the material was submitted to microscopic analysis, how many blocks were generated from the specimen (five blocks), and how many fragments were placed in each block (blocks 1 and 2 contain six fragments and the other blocks contain one fragment each): “Approximately circular skin fragment measuring 3.0 × 2.5 × 0.8 cm showing, in the center, a brownish pigmented lesion with irregular borders, with blackened areas measuring 1.8 × 1.2 cm and 0.2 cm from the closest margin.

All the material was submitted for histological analysis. Legend: 1: Extremity (6F); 2: Extremity (6F); 3: Central portion (1F); 4: Central portion (1F); 5: Central portion (1F)”.

**Recommendation 4**: Whenever possible, include all the material sent in case of lesions suspected of melanoma. Clinicians and surgeons are advised to verify whether this recommendation was followed by carefully reading the macroscopy section of the anatomopathological report.

## Microscopy

**Figure 6:**
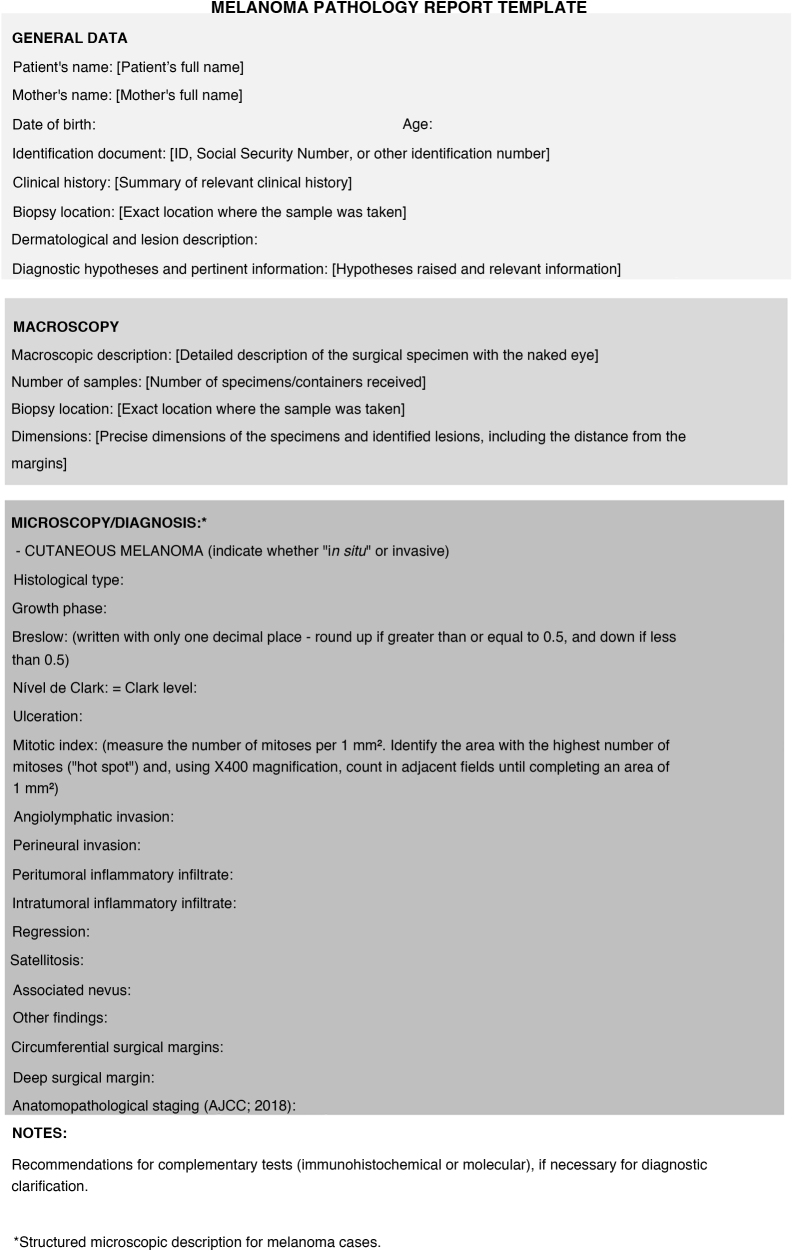
Example of a structured report. It is important to note that some cases do not require notes and services have the autonomy to adapt the structure by inverting the order or merging, for example, microscopy with diagnosis.

**Figure 7:**
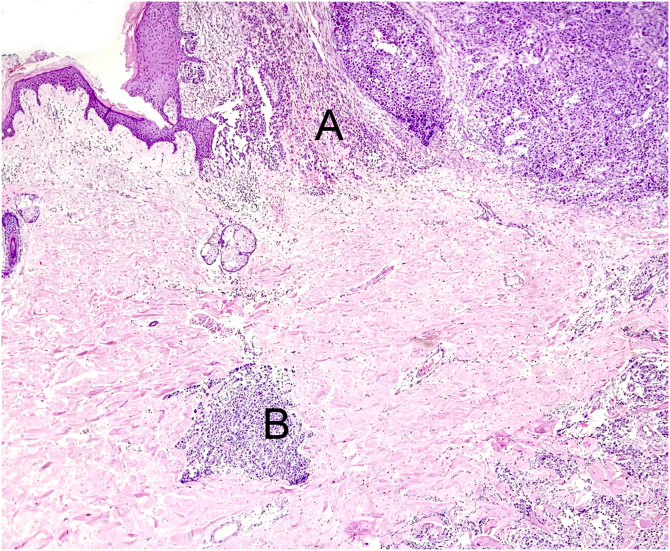
The histological section (Hematoxylin & eosin ×40) shows the primary melanoma in vertical growth (A) and microsatellite (B) in the dermis. The microsatellite is characterized by discontinuous tumor proliferation, adjacent to the main tumor, permeated by normal tissue, without findings of inflammatory infiltrate or fibrosis that could suggest tumor regression.

**Figure 8:**
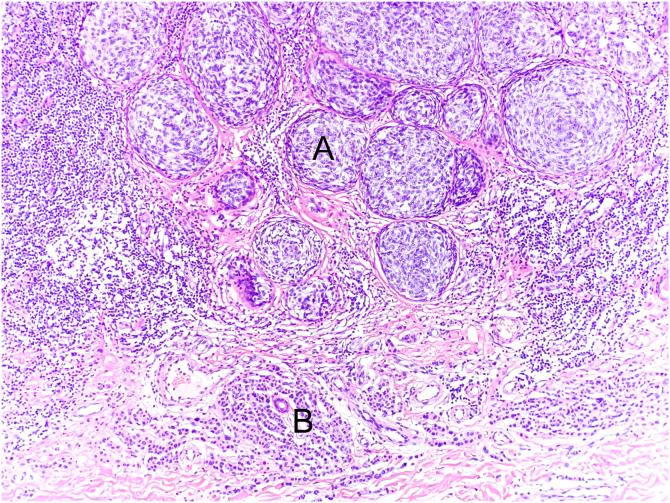
The histological section (Hematoxylin & eosin ×40) shows melanoma (A) associated with intradermal melanocytic nevus (B). The melanoma is characterized by nests of atypical melanocytic cells without signs of maturation and exhibiting moderate nuclear pleomorphism (A). Below (B) small melanocytic cells can be observed, scattered throughout the dermis, exhibiting scarce cytoplasm and no cytological atypia.

Following current international recommendations,^10^, ^11^ the melanoma excision report should contain the following parameters: histological type; Breslow index (maximum tumor thickness) measured in millimeters, considering only one decimal place (after the period) and drawing a straight line between the deepest tumor cell and the most superficial cell of the granulosa layer; presence/absence of ulceration; mitotic index in the “hot spot” with the mitosis count per 1 mm^2^ area; angiolymphatic invasion; perineural invasion; presence/absence of satellitosis; presence/absence of associated nevus; surgical margin status and anatomopathological staging (Figure 6, Figure 7, Figure 8). Margin widening is part of melanoma treatment regardless of staging, so it is not necessary to measure the tumor distance in relation to the margin. The main parameters for melanoma staging are the Breslow index and the presence of ulceration, which define cutaneous melanoma staging.^10^, ^11^ A recent Brazilian study also demonstrated a significant association between these two parameters and the result of the sentinel lymph node and patient survival with invasive cutaneous melanoma.^22^ The following items: Clark level, growth phase, presence/absence of peri- and intratumor inflammatory infiltrate and presence/absence of regression have been losing value in recent years, and are no longer mandatory in the report.^11^

Ulceration is defined by the combination of the following microscopic findings: complete loss of epidermis (including the absence of stratum corneum and basement membrane); evidence of reactive changes (e.g., presence of fibrino-leukocyte infiltrate); and thinning/effacement or reactive hyperplasia of the adjacent epidermis in the absence of recent trauma or surgical procedure.^11^ If the ulceration is secondary to a prior biopsy, it should not be reported and/or considered for staging purposes.^10^, ^11^ Unlike invasive melanomas, the presence of ulceration does not alter the staging of cases of melanoma “*in situ*”.^10^, ^11^ Furthermore, it is suggested that ulceration extent is a more accurate predictor of survival than the simple presence of ulceration in cases of invasive cutaneous melanomas.^23^, ^24^

Considering the latest editions of the World Health Organization classification of skin tumors, the histological subtype definition was redefined considering the carcinogenesis pathways and the most frequently mutated genes in each histological subtype. In general terms, the four most common subtypes (superficial spreading, lentigo malignant melanoma, acral and nodular) were redivided into: melanoma associated with chronic sun exposure/high cumulative sun damage (which includes cases of lentigo malignant melanoma and some cases of nodular melanomas), melanoma associated with intermittent sun exposure/low cumulative sun damage (which includes cases of superficial spreading melanoma and some cases of nodular melanomas) and acral melanomas for which the relationship with sun exposure is not yet clear. Some less common types, such as the Spitz melanoma group, may require molecular confirmation for correct diagnosis.^6^

Regarding the evaluation of the lymphocytic inflammatory infiltrate, it can be classified as absent, present but not active (“non-brisk”), or active (“brisk”). To be considered active, the presence of lymphocytes among the tumor cells is not necessary, since the inflammatory cells may only be on the lesion periphery (border). Although it is an optional item in the report, the prognostic value of lymphocytes permeating or infiltrating melanoma cells remains under discussion.^25^, ^26^

Aiming at performing the anatomopathological diagnosis of melanocytic lesions in diagnostic categories based on risk stratification, rather than on the classic histological diagnosis, the system that receives the English name “The Melanocytic Pathology Assessment Tool and Hierarchy for Diagnosis (MPATH-Dx)” was created in 2014.^27^ Version 2.0 of this system was published in 2023, which shows four diagnostic classes: class I for lesions with a very low risk of progression to invasive melanoma (e.g., dysplastic nevi with low-grade cytological atypia), class II for lesions with a low risk of progression to invasive melanoma (e.g., dysplastic nevi with high-grade cytological atypia and melanoma “*in situ*”), class III for lesions with a low risk of metastasis (e.g., melanoma stage pT1a) and class IV for lesions with a moderate to high risk of metastasis (e.g., melanomas stage ≥ pT1b).^9^ It is believed this classification system would increase diagnostic agreement between services, reducing academic discussions that are not relevant to patient monitoring but are often not understood by the lay public. To cite an example, it would be the differentiation between melanoma “*in situ*” and dysplastic nevus with high-grade cytological atypia since both lesions would be classified as class II of MPATH-Dx^9^ and patients would have to undergo surgical margin expansion. Categorization according to the classes of this system remains optional and scarcely used in the medical community in the studied country. Some barriers to the use of this system would be the establishment of a conduct that is seen with a more comprehensive view of the patient and not only based on histopathological findings, as well as the indication of more aggressive conduct by the system, such as margin extention in Spitz nevi and cellular blue nevi, for example.^9^

## Immunohistochemistry

It is a misconception to think that every case of melanocytic lesion should be submitted to immunohistochemical study. Therefore, the use of such technique is indicated for: I) Lineage confirmation in cases of poorly differentiated melanomas; II) Cell proliferation index assessment (Ki-67); III) Research of markers related to specific molecular alterations (ALK, ROS, BAP1, BRAF, NTRK, and p16).^6^ It is worth noting, however, that there is currently no single, validated, known, and long-standing marker to differentiate benign and malignant melanocytic lesions. Furthermore, the results of immunohistochemical markers should always be interpreted together with histopathological findings.

For highly pigmented lesions, special techniques can be used: the use of magenta chromogen in the immunohistochemical reaction (preferred method), counterstaining with Giemsa, and depigmentation with hydrogen peroxide are some of the options.^28^, ^29^ It is important to note that depigmentation with hydrogen peroxide must be performed very carefully because there is a high risk of damaging the material, making the reaction unfeasible.

For cases that are difficult to interpret, some melanocytic markers are very sensitive (e.g., MITF, SOX10 and S100) and specific (e.g., MART1); they are used to detect cell density and neoplastic growth patterns when it is not possible to do that with Hematoxylin & eosin staining (due to coexisting inflammation, artifact, etc.). In desmoplastic melanomas, the most sensitive antibody is the one that detects the S-100 protein.^6^ HMB-45 (Human Melanoma Black-45) is a valuable marker for the differential diagnosis between nevus and melanoma since cell maturation is identified through a change in color gradient in benign lesions, which is stronger in the upper portions and negative in the deep portion. HMB-45 is also useful in differentiating between nevus and subcortical metastases in sentinel lymph node evaluation.^30^ Careful analysis should also be performed for the p16 protein, encoded by the CDKN2a tumor suppressor gene, which acts by slowing cell division through cell cycle progression inhibition. Loss of p16 nuclear expression assessed in immunohistochemistry is associated with significantly increased tumor cell proliferation, which may indicate a more aggressive tumor.^30^

The Ki-67 antibody marks any cell that is not at rest (G0 phase of the cell cycle), that is, it is positive in all cells in the G1, S, G2 phases, and in mitosis. Therefore, it is not a specific marker for melanocytes. In cases with dense inflammatory infiltrate, double staining of MART-1 using the magenta chromogen (cytoplasmic staining) and diaminobenzidine for K-i67 (nuclear staining) is a useful tool if there is interpretive doubt. It is also noteworthy that nevi in adult patients generally do not show mitoses and exhibit low cell proliferation characterized by rare Ki-67-positive cells and no positive cells in the deep portion of the lesion. To date, there is no cutoff number for the percentage of Ki-67-positive cells that differentiates benign from malignant melanocytic lesions considering this marker alone.^20^, ^31^

The PRAME antibody (PRAME-PReferentially expressed Antigen in MElanoma) is a recent and more efficient marker for differentiating nevi from melanomas, with preferential staining in melanoma in relation to benign lesions. However, it is a marker whose positivity does not overlap with morphological findings since there is no defined pattern in doubtful cases that are difficult to interpret diagnostically (intensity and extent of staining).^32^ In addition, there is more than one clone of this antibody on the market, making it difficult to draw conclusions regarding the experience of different services and published articles.

**Recommendation 5:** Immunohistochemical studies are not essential for the microscopic diagnosis of melanoma, being reserved for selected cases, indicated by the pathologist in the report, often through comments and notes.

## Perioperative examination

The frozen section examination is one of the types of perioperative examination and consists of the microscopic analysis of a specimen during the patient's surgery. It is an indicated and fundamental tool in the management of different clinical scenarios, providing valuable information to the surgeon, such as the diagnosis of malignancy or the presence of neoplasia in a margin, allowing therapeutic conduct to be taken during the surgical procedure.^33^ Briefly, it consists in submitting a representative tissue fragment to freezing, allowing it to be cut very thinly, with subsequent staining. In this examination, a limited microscopic visualization is performed, allowing a preliminary (not definitive) diagnosis during the surgical procedure. However, in the scenario of melanocytic lesions, freezing a tumor suspected of melanoma is not appropriate and may put the patient's accurate diagnosis at risk.^11^

Below are some reasons: I) The act of freezing the material generates artifacts in the tissue that impairs morphology. Melanocytic lesions that are difficult to interpret often require serial sections for accurate evaluation, and correct microscopic interpretation is not possible due to the less clear visualization provided by the frozen material. II) It is not possible to accurately evaluate cytological atypia in frozen sections.^34^ III) Melanomas often have unclear limits and may present with “skip lesions”^35^, preventing margin evaluation in these cases. IV) Even in histological sections after adequate fixation, it may be difficult to microscopically differentiate melanocytic proliferation secondary to solar damage from lentigo maligna, and this differentiation cannot be properly performed in the histological evaluation of the frozen section.^35^ V) Additionally, the freezing process may damage the material and even impair subsequent immunohistochemical evaluation.

Considering that perioperative examination is contraindicated, any type of frozen section examination, including Mohs micrographic surgery, to define margins or diagnose a primary melanocytic lesion of the skin has to be avoided. The gold standard for evaluating surgical margins is the microscopic examination of slides stained with Hematoxylin & eosin, after correct fixation and histological processing. Performing a perioperative immunohistochemical study is not a validated technique and is not recommended. Freezing a tumor suspected of melanoma is not appropriate and may jeopardize the patient's diagnosis.^8^

**Recommendation 6**: Perioperative examination is contraindicated for both diagnosis and margin evaluation of melanocytic lesions.

## Sentinel lymph node

The treatment of melanoma is a rapidly evolving field, and the role of the sentinel lymph node has been debated in certain clinical settings but remains an important tool for disease staging and prognosis. Therefore, appropriate handling, macroscopy, and microscopic evaluation of the sentinel lymph node are mandatory to guide appropriate care for the melanoma patient. Frozen section examination of the sentinel lymph node is also contraindicated because the manipulation required to perform the perioperative examination may reduce the procedure sensitivity.^11^, ^36^

For melanoma examination, careful handling of the sentinel lymph node is recommended to avoid damage to the lymph node capsule. The lymph node should be measured, sectioned, and submitted in its entirety for histological processing and subsequent microscopic evaluation by a pathologist.

Several parameters have been reported for the microscopic evaluation of tumor burden in the sentinel lymph node of a patient with melanoma, including microanatomic location, largest deposit axis, tumor depth, surface area and surface area ratio, volume, size, and cell count.^37^ The minimum report should include both dimensions of the deposit and the presence of extranodal extension.

If more than one lymph node is sent for analysis, both lymph nodes should be analyzed and their identification and description should be kept separate.

**Recommendation 7**: Perioperative examination is contraindicated for sentinel lymph node evaluation in cases of cutaneous melanoma.

## Molecular assessment

The molecular tests currently applied in the routine of patients with melanoma can be divided into germline and somatic.

The main germline molecular alterations related to the risk of developing melanoma are alterations in the *CDKN2a, CDK4, MC1R, BAP1, TERT, MITF, PTEN* genes. In general, the suspected patient is one with a personal and/or family history of melanoma, multiple melanomas, and cancers in the family such as pancreatic, kidney, breast, uveal, mesothelioma and astrocytoma. It is recommended that the investigation be conducted together with an oncologist/geneticist for correct counseling and additional decisions on testing the individual/family.

Somatic alterations, that is, present in neoplastic cells only, can be divided into two large groups: those for therapeutic decision-making purposes (predictive) and alterations that may be useful for diagnostic purposes.

Testing for mutations involving codon 600 of the *BRAF* gene is necessary to establish therapeutic decisions in patients with stage 3 and 4 melanoma. Its role in high-risk stage II has been increasing. The presence of this mutation can be tested using different techniques, including immunohistochemical studies using the VE1 antibody clone, and techniques such as PCR and Next Generation Sequencing (NGS). The sample to be tested is a sample from histological processing, paraffin-embedded and representative of the tumor, which may be from the primary lesion or metastasis. The sample must be evaluated by a pathologist to ensure that it represents a sufficient quantity of tumor cells for testing. The molecular test success depends directly on the quality of the genetic material of the lesion in the paraffin block, which depends, among other factors, on how the sample was fixed and packaged.

There are many tests described in the literature with potential diagnostic usefulness for melanocytic lesions, in defining whether a lesion is benign or malignant. Among the tests, there are gene expression tests, tests that evaluate losses and gains in genomic regions such as Fluorescent In-Situ Hybridization (FISH) and Comparative Genomic Hybridization (CGH) tests. None of these tests alone, to date, is indicated for use to define between benign and malignant lesions. A thorough morphological analysis supported by solid clinical-pathological correlation remains, to date, the main tool in the diagnosis of melanocytic lesions. The NGS test, by identifying molecular alterations characteristic of any of the nine distinct molecular pathways of melanocytic lesions, can help in the diagnostic definition of these lesions.

**Recommendation 8:** Molecular testing to detect mutations in the *BRAF* gene is recommended in the scenario of patients with high-risk stage II, 3 and 4 melanoma, using a sample with adequate neoplastic representation and a molecular technique duly validated by the laboratory.

## Conclusions

For an accurate melanoma diagnosis, a close clinical-pathological correlation is essential. Excisional biopsy with exigous margins (1-3 mm) is the preferred approach, and the specimen should be immediately submerged in buffered formalin, and the fixation time should not exceed 72 hours. Diagnosis continues to be made through complete inclusion of the lesions and evaluation using Hematoxylin & eosin staining. Complementary studies (immunohistochemical and molecular) are only applied to a small percentage of cases. The perioperative examination is expressly contraindicated for the diagnosis or margin evaluation of suspicious melanocytic lesions, as well as for the evaluation of the sentinel lymph node in cases of cutaneous melanomas.

## Financial support

Novartis company funded an in-person meeting of specialist pathologists, without any type of interference in the content of this study.

## Authors' contributions

José Cândido Caldeira Xavier Junior: Design and planning of the study; collection of data, or analysis and interpretation of data; drafting and editing of the manuscript or critical review of important intellectual content; effective participation in research orientation; critical review of the literature; approval of the final version of the manuscript.

Karina Munhoz de Paula Alves Coelho: Design and planning of the study; collection of data, or analysis and interpretation of data; drafting and editing of the manuscript and critical review of important intellectual content; critical review of the literature; approval of the final version of the manuscript.

Mariana Petaccia de Macedo: Design and planning of the study; collection of data; drafting and editing of the manuscript, collection, analysis and interpretation of data; critical review of the literature; approval of the final version of the manuscript.

Rute Facchini Lellis: Design and planning of the study; drafting and editing of the manuscript and critical review of important intellectual content; graphic design; approval of the final version of the manuscript.

Nathanael de Freitas Pinheiro Junior: Design and planning of the study; critical review of important intellectual content; approval of the final version of the manuscript.

Robledo Fonseca Rocha: Design and planning of the study; critical review of important intellectual content; critical review of the literature; approval of the final version of the manuscript.

## Conflicts of interest

None declared.
